# A Toxin-Conjugated Recombinant Protein Targeting gp120 and gp41 for Inactivating HIV-1 Virions and Killing Latency-Reversing Agent-Reactivated Latent Cells

**DOI:** 10.1128/mbio.03384-21

**Published:** 2022-01-18

**Authors:** Xinling Wang, Wei Xu, Zezhong Liu, Yanling Wu, Qian Wang, Miao Cao, Tianlei Ying, Na He, Lu Lu, Shibo Jiang

**Affiliations:** a Shanghai Public Health Clinical Center, Key Laboratory of Medical Molecular Virology (MOE/NHC/CAMS), School of Public Health and The Key Laboratory of Public Health Safety of Ministry of Education, School of Basic Medical Sciences, Shanghai Institute of Infectious Disease and Biosecurity, Fudan University, Shanghai, China; b Key Laboratory of Reproduction Regulation of National Population and Family Planning Commission, The Shanghai Institute of Planned Parenthood Research, Institute of Reproduction and Development, Fudan University, Shanghai, China; Columbia University Medical College

**Keywords:** HIV-1, virus inactivator, toxin-conjugated recombinant protein, HIV-1 latent infection, shock and kill

## Abstract

Application of the combination antiretroviral therapy (cART) has reduced AIDS to a manageable chronic infectious disease. However, HIV/AIDS cannot be cured because of the presence of latent reservoirs, thus calling for the development of antiretroviral drugs that can eliminate latency-reversing agent (LRA)-activated HIV-1 virions and latent cells. In this study, we conjugated a small-molecule toxin, DM1, to a gp120-binding protein, mD1.22, a mutated CD4 domain I, and found that mD1.22-DM1 could inactivate HIV-1 virions. However, it could not kill LRA-activated latent cells. We then designed and constructed a dual-targeting protein, DL35D, by linking mD1.22 and the single-chain variable fragment (scFv) of a gp41 NHR-specific antibody, D5, with a 35-mer linker. Subsequently, we conjugated DM1 to DL35D and found that DL35D-DM1 could inhibit HIV-1 infection, inactivate HIV-1 virions, kill HIV-1-infected cells and LRA-reactivated latent cells, suggesting that this toxin-conjugated dual-targeting recombinant protein is a promising candidate for further development as a novel antiviral drug with potential for HIV functional cure.

## INTRODUCTION

Multiple antiviral drugs against HIV-1 that caused AIDS (1) have been developed and approved (2), including reverse transcriptase inhibitors, protease inhibitors, and entry inhibitors. The administration of combination antiretroviral therapy (cART) has resulted in significant reduction of morbidity and mortality. However, no approved therapeutics can entirely eliminate HIV-1 infection since HIV-1 is able to integrate into the host cell genome and establish a latent HIV-1 reservoir, the main barrier to an HIV cure (2, 3). This calls for the development of novel antivirals to eliminate the latency-reversing agent (LRA)-activated HIV virions and latently HIV-infected cells.

To achieve a cure, or functional cure, of HIV/AIDS, some research groups have proposed immune-based strategies, antibodies, gene editing, T-cell vaccine, and immunotoxin (4). One approach, called “shock and kill,” uses an LRA to activate the virions in the latently infected cells, which are then expected to be killed by the patient's immune system (5). However, clinical trials revealed that treatment with LRA could, indeed, activate viral RNA expression, but still not eliminate the reactivated HIV-infected cells by resort of the immune system (6). In addition, reactivated latent-infected cells in patients receiving antiretroviral therapy were rarely cleared by apoptosis (7). Commonly, no viral proteins are expressed on HIV latent-infected cells (5). Using LRA reactivation, newly expressed Env on the cell surface could be recognized by the human immune system, and it could also act as the target of antiviral drugs.

**FIG 1:**
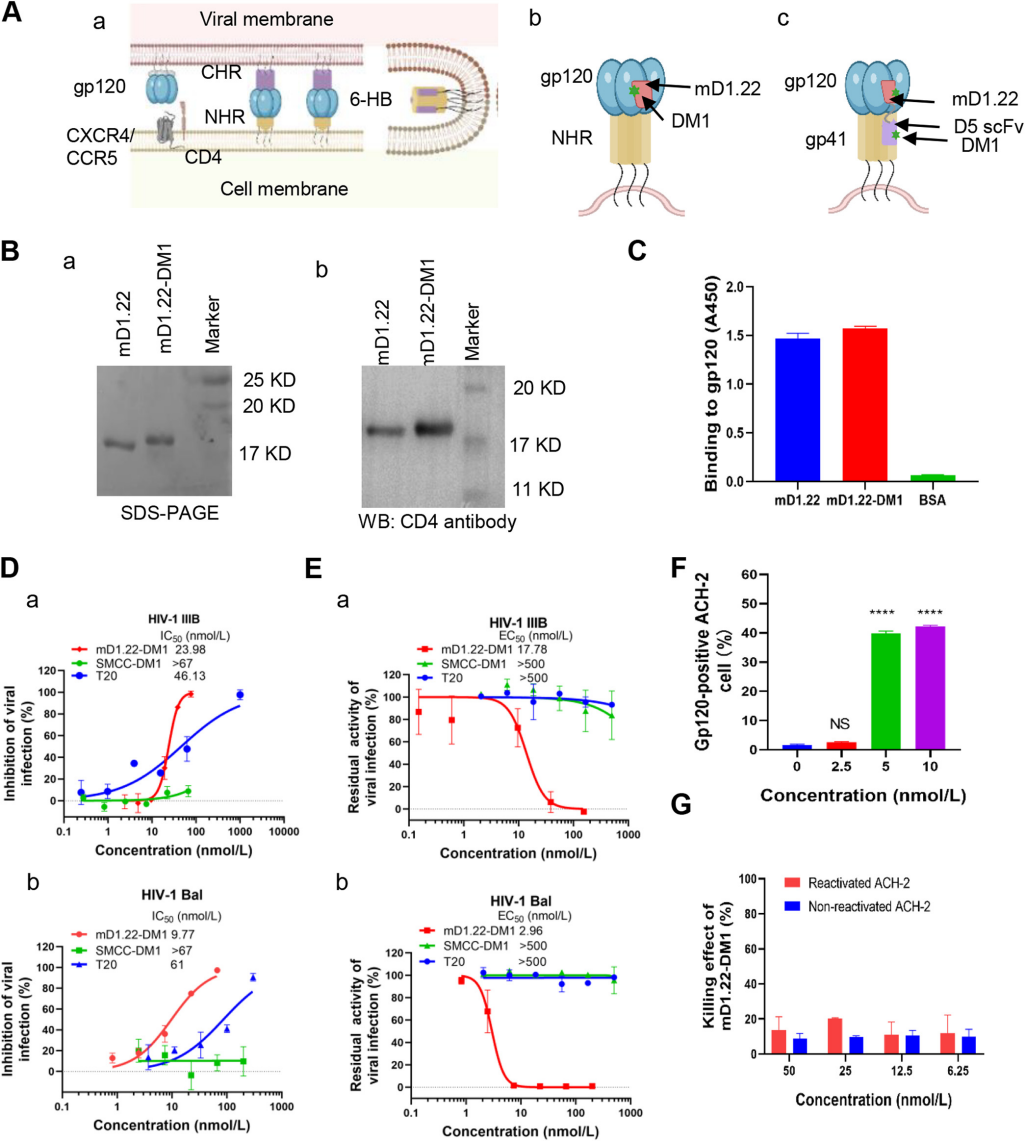
Activity of toxin-conjugated protein mD1.22-DM1 *in vitro*. (A) Cartoon of the membrane fusion process and the mechanisms of mD1.22-DM1 and DLD-DM1. (a) After HIV-1 gp120 binds to the receptor CD4 molecule and then a co-receptor, CXCR4 or/CCR5, on the target cell, serial conformational changes occur in gp120 and gp41, including the exposure of the gp41 NHR and formation of NHR-trimer, which then interacts with 3 CHR molecules to form a 6-HB, resulting in virus-cell membrane fusion. (b) mD1.22-DM1 binds to gp120 through its the mD1.22 portion and then delivers DM1 to the target cell. (c) DLD-DM1 binds to gp120 through its mD1.22 portion and gp41 NHR domain via its D5 scFv portion, resulting in the release of DM1 to the target cell. (B) Analysis of mD1.22 and mD1.22-DM1 by SDS-PAGE (a) and Western blot using a rabbit anti-CD4 polyclonal antibody (b). (C) Detection of the binding activity of mD1.22-DM1 to HIV-1 gp120 by ELISA. The protein mD1.22 and toxin-conjugated protein mD1.22-DM1 were coated onto the wells of a 96-well plate at 5 μg/ml, respectively, followed by addition of gp120 at 3 μg/ml. The binding activity was determined by using mouse anti-gp120 sera. (D) Inhibitory activity of mD1.22-DM1 against infection of HIV-1 laboratory-adapted strains IIIB (a) and Bal (b). The red, green, and blue curves represent the HIV-1 inhibitory activity of mD1.22-DM1, SMCC-DM1, and T20 peptide, respectively. (E) The inactivation activity of mD1.22-DM1 against HIV-1 laboratory-adapted strain IIIB (a) and Bal (b). The red, green, and blue curves represent the HIV-1 inactivation activity of mD1.22-DM1, SMCC-DM1, and T20 peptide, respectively. (F) The proportion of gp120-positive ACH-2 cells 12 h post-stimulation with romidepsin. ACH-2 cells were treated with romidepsin at different concentrations. Then the cells were incubated with the HIV-1 gp120-specific human antibody N6. The proportion of gp120-positive ACH-2 cells was detected with FITC-labeled goat anti-human IgG antibody using flow cytometry. (G) The killing effect of mD1.22-DM1 on LRA-reactivated and nonreactivated ACH-2 cells. One-way ANOVA was used in the statistical analysis. **** and NS mean *P* < 0.0001 and no statistical significance, respectively.

The envelope protein of HIV-1 is a complex of gp120 and gp41. To infect the target cell, gp120 binds to cell receptor CD4 molecule and co-receptor CXCR4 or CCR5, inducing conformational change of gp120. Following this, the fusion peptide (FP) of gp41 inserts into target cells, and 3 NHR molecules interact with each other to form an inner NHR-trimeric helix. Then, 3 CHR molecules bind to the NHR trimer to form a six-helix bundle (6-HB), which brings viral and target cell membranes into close proximity for fusion (8–10) (Fig. 1Aa). Based on the CD4 receptor, a recombinant protein, CD4-PE40, which consists of the first 178 amino acids of CD4 molecules and domains II and III of pseudomonas exotoxin A (PE40) (11), displayed specific toxicity to HIV-1-infected cells (11, 12). However, it was revealed that the CD4 molecule in the recombinant protein could have adverse effects on the otherwise normally functioning immune system, potentially enhancing HIV-1 infection in CD4-CCR5^+^ cells (13). ACH-2 cells, a cellular model used to study HIV latency, derived from an HIV-infected A3.01 (CD4^+^ T) cells, could be reactivated with latent-reversing agents (14, 15). In a previous report, the number of HIV-1 Env-positive ACH-2 cells is correlated with the concentration of LRA tested (16). Consequently, it is unknown whether CD4-PE40 could kill the ACH-2 cells that are not fully reactivated by LRA. Moreover, CD4-PE40 treatment showed no reduction of HIV-1 RNA load in plasma and provirus level in PBMCs (17). It could also induce anti-PE antibodies in some patients (18). Therefore, it is essential to develop an efficient toxin-conjugated protein with better specificity and safety than CD4-PE40 to finally achieve an HIV-1 functional cure.

Accordingly, in this study, we first constructed a toxin-conjugated recombinant protein, mD1.22-DM1 (Fig. 1Ab), which consists of mD1.22 (a modified single CD4 domain), SMCC (an uncleavable linker), and DM1 (a potent microtubule-disrupting agent that can promote apoptosis by interfering with mitosis [19]). The mD1.22, a cavity-altered CD4 domain I with high affinity for gp120, good stability and solubility, exhibits broad and potent neutralizing activity against HIV-1 infection (20). SMCC-DM1 was conjugated to an HER2 antibody to generate an anticancer drug, Emtansine (formerly called Trastuzumab-DM1), which has been approved for clinical treatment of invasive HER2-positive breast cancer (21). We found that mD1.22-DM1 could inactivate HIV-1 virions, but not LRA-activated latent cells.

Our previous study has shown that combining mD1.22 with a gp41 NHR-binding antibody D5 single-chain variable fragment (scFv) exhibits synergistic anti-HIV-1 effect and that D5 scFv could enhance mD1.22-mediated HIV-1 inactivation (22). Based on these findings, we designed and built a dual-targeting protein, DL35D, by linking mD1.22 and D5 scFv with a 35-mer linker. We next conjugated DM1 to DL35D to produce a toxin-conjugated dual-targeting protein, DL35D-DM1 (Fig. 1Ac). We found that DL35D-DM1 could effectively inhibit HIV-1 infection, inactivate HIV-1 virions, and kill HIV-1-infected cells and LRA-reactivated latent cells, suggesting that it is a promising candidate for development as a novel antiviral drug with potential as an HIV functional cure.

## RESULTS

### mD1.22-DM1 exhibits inhibitory and inactivation activity against divergent HIV-1 strains, but no killing effect on LRA-reactivated latent-infected cells.

The recognition of gp120 by CD4 and CXCR4 or CCR5 triggers virus fusion and entry into the target cell (Fig. 1Aa). An engineered cavity-altered single-domain CD4 protein, mD1.22, with high gp120-binding affinity, is a potent HIV-1 entry inhibitor (20). After the mD1.22 component in mD1.22-DM1 binds to gp120, we propose that its DM1 component could be delivered to the cells to disrupt the microtube system (23–25) (Fig. 1Ab). Therefore, we expressed and purified mD1.22 protein and then conjugated DM1 to it to form an mD1.22-DM1 conjugate. According to SDS-PAGE, the position of mD1.22-DM1 in the gel was slightly higher than that of mD1.22 (Fig. 1Ba), suggesting that some DM1 molecules were conjugated to mD1.22. In Western blot, both mD1.22 and mD1.22-DM1 were recognized by rabbit anti-human CD4 polyclonal antibody (Fig. 1Bb). We then conducted ELISA to determine whether mD1.22-DM1 could interact with gp120. As shown in Fig. 1C, mD1.22-DM1 was, indeed, bind with HIV-1 gp120 as strongly as mD1.22. Subsequently, we tested mD1.22-DM1 for its inhibitory activity against infection of HIV-1 laboratory strain IIIB (X4) and Bal (R5). As shown in Fig. 1D, mD1.22-DM1 displayed potent inhibitory activity against HIV-1 IIIB and Bal with the half maximal inhibitory concentration (IC_50_) values of 23.98 and 9.77 nmol/liter, respectively, which was more effective than the HIV-1 fusion inhibitory peptide T20 (IC_50_: 46 and 61 nmol/liter), while the control toxin molecule SMCC-DM1 did not inhibit HIV-1 infection at the concentration up to 67 nmol/liter. Our previous study showed that mD1.22 could bind gp120 and inactivate laboratory-adapted and primary HIV-1 virions (22). Similarly, mD1.22-DM1 also exhibited inactivation activity against cell-free HIV-1 IIIB and Bal particles with median effective concentration (EC_50_) values of 17.78 and 2.96 nmol/liter, respectively. In contrast, DM1 and T20 had no inactivation activity with a concentration up to 500 nmol/liter (Fig. 1E). These results indicate that mD1.22-DM1 can efficiently inhibit HIV-1 infection and inactivate cell-free HIV-1 virions.

Next, we tested the killing effect of mD1.22-DM1 on LRA-reactivated latent ACH-2 cells using romidepsin as an LRA. It was reported that romidepsin, a histone deacetylase inhibitor (HDACi), could efficiently reactivate latent HIV-1 *in vivo* (26). It was also reported that romidepsin could reactivate ACH-2 cells by inducing 54% of ACH-2 cells expressing Env protein 24 h post-stimulation at the concentration of 3 nmol/liter (16). Here, we found that romidepsin could significantly reactivate latent HIV-1 at the concentration of 5 nmol/liter, as shown by the increased expression of p24 in the cell lysate (Fig. S1) and the enhanced expression of Env on the surface of ACH-2 cells (Fig. 1F). Romidepsin induced about 40%, 37% and 1% of ACH-2 cells expressing Env protein 12 h post-stimulation at the concentration of 10, 5 and 2.5 nmol/liter, respectively (Fig. S2). We then used romidepsin to reactivate ACH-2 cells at the concentration of 5 nmol/liter and found that mD1.22-DM1 exhibited no significant killing effect on LRA-reactivated ACH-2 cells at the concentration up to 50 nmol/liter (Fig. 1G). Therefore, when its target is the CD4-binding site on gp120, mD1.22-DM1 cannot kill LRA-reactivated ACH-2 cells.

10.1128/mbio.03384-21.1 FIG S1 The relative p24 production of LRA-reactivated ACH-2 cells. The ACH-2 cells were simulated with romidepsin at different concentrations for 12 hours. The p24 level in supernatants was tested by ELISA. Download FIG S1, PDF file, 0.1 MB. Copyright © 2022 Wang et al. 2022 Wang et al. https://creativecommons.org/licenses/by/4.0/ This content is distributed under the terms of the Creative Commons Attribution 4.0 International license.

10.1128/mbio.03384-21.2 FIG S2 Detection of the percentage of simulated gp120-positive ACH-2 cells using flow cytometry 12h post-simulation with romidepsin. The grey curve represents un-simulated ACH-2 cells. The red curves represent ACH-2 cells simulated with romidepsin at the concentrations of 2.5 nM (A), 5 nM (B), and 10 nM (C), respectively. HIV-1 gp120-specific human antibody N6 was used in this experiment. Download FIG S2, PDF file, 0.2 MB. Copyright © 2022 Wang et al. 2022 Wang et al. https://creativecommons.org/licenses/by/4.0/ This content is distributed under the terms of the Creative Commons Attribution 4.0 International license.

### Construction, expression, and detection of the dual-targeting recombinant protein.

**FIG 2:**
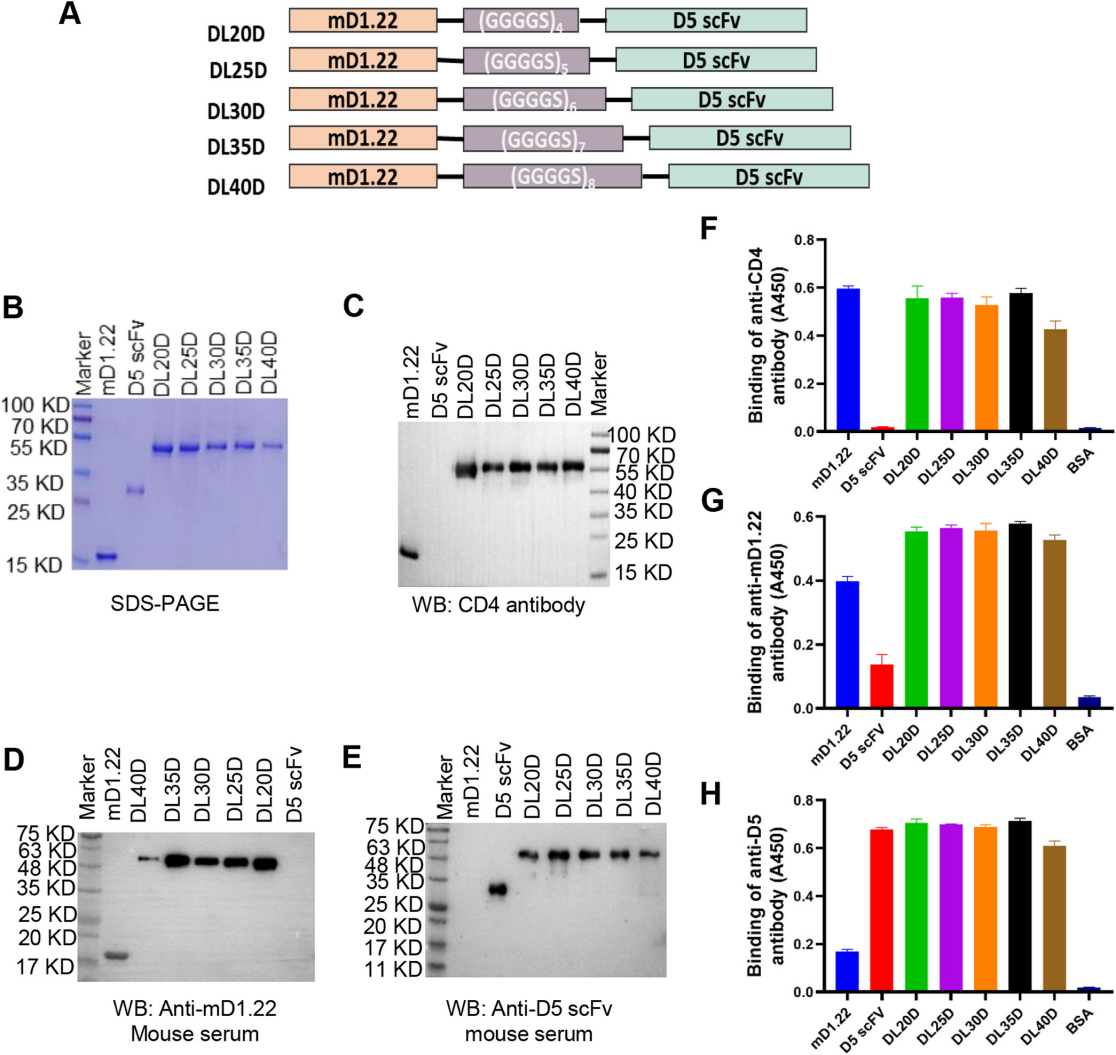
Expression and verification of dual-targeting recombinant proteins, DLDs. (A) Construction strategy for dual-targeting recombinant proteins, DLDs. A DLD is comprised of mD1.22 and D5 scFv linked with a flexible linker (GGGGS)n, in which “n” means 4, 5, 6, 7, or 8. Analysis of DLDs with SDS-PAGE (B). Detection of DLDs with Western blot using a rabbit anti-human CD4 polyclonal antibody (C), mouse anti-mD1.22 serum (D), and mouse anti-D5 scFv serum (E). Detection of binding of DLDs in ELISA with anti-CD4 antibody (F), mouse anti-mD1.22 antibody (G), and mouse anti-D5 antibody (H), respectively.

Since the NHR of gp41, exposed transiently during the membrane fusion process, could serve as a conserved target for the development of HIV-1 entry inhibitors, we linked the gp120-binding protein mD1.22 and a gp41-targeting single-chain fragment (scFv) of an NHR-specific antibody D5 with a flexible linker (GGGGS)n, in which n means 4, 5, 6, 7 or 8, to construct a dual-targeting recombinant protein, mD1.22-linker-D5 scFv (DLDs) (Fig. 2A). (GGGGS)n were commonly used linkers with high flexibility, allowing the two functional domains linked move freely (27). DLDs proteins were expressed in Expi293 cells, purified with affinity chromatography, and verified by SDS-PAGE and Western blot. With different linker length, expected molecular weight of DL20D, DL25D, DL30D, DL35D, and DL40D were about 38.7, 39.0, 39.3, 39.7, and 40.1 kDa, respectively (Fig. 2B). The yield of DL20D and DL35D is higher than other DLDs, while that of DL40D is the lowest. We could obtain about 1 mg of DL20D and DL35D from 100 ml of Expi293 cells, while only about 0.2 mg of DL40D from 100 ml of Expi293 cells. Similar to mD1.22, all of these proteins reacted with rabbit anti-human CD4 polyclonal antibody and anti-mD1.22 mouse serum (Fig. 2C and D), whereas no band was shown in the line where D5 scFv was loaded. These DLDs also reacted with anti-D5 scFv mouse serum (Fig. 2E). Similarly, DLDs were be recognized by anti-CD4 antibody and anti-mD1.22 mouse serum, as well as anti-D5 scFv mouse serum, as determined by ELISA (Fig. 2F to H). These results suggest that DLDs comprised of mD1.22 and D5 scFv were successfully expressed.

### Bifunctional protein DLDs bind with gp120 and gp41 to inhibit 6-HB formation.

**FIG 3:**
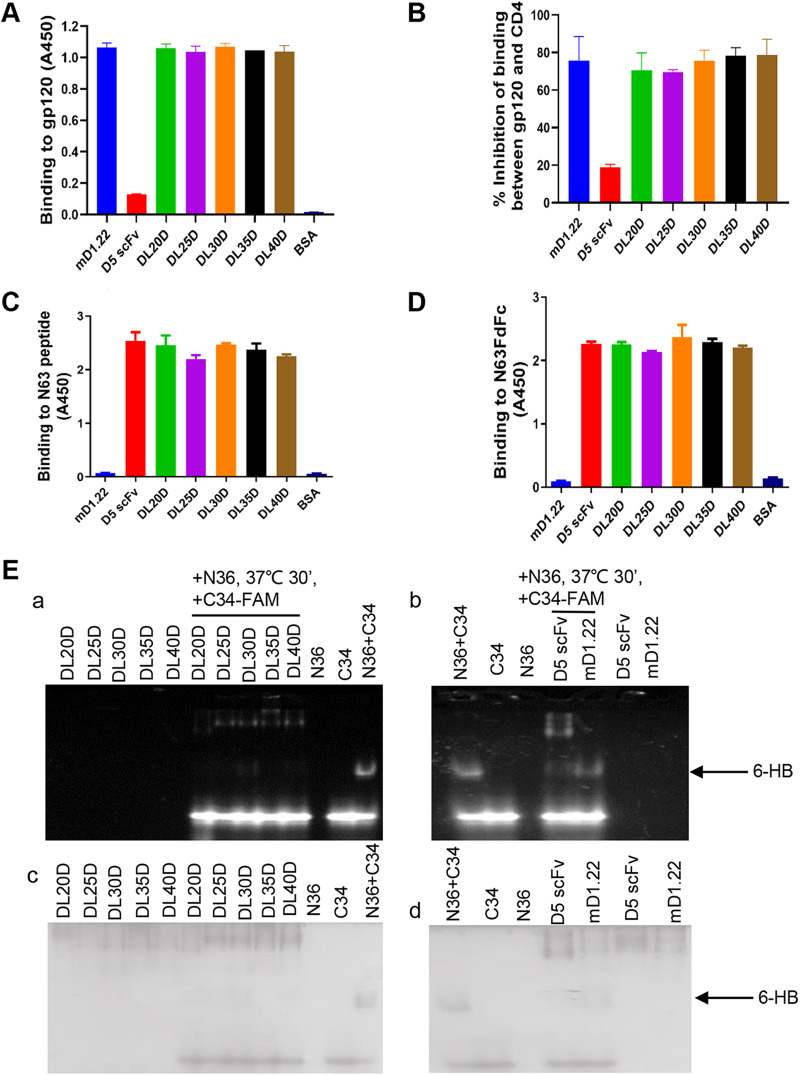
Detection of the antiviral mechanism of DLDs. (A) Binding activity of DLDs with gp120 was measured with ELISA using mouse anti-His-Tag monoclonal antibody. (B) Inhibitory activity of DLDs on the binding of gp120 and CD4 receptor on the target cell was measured with cell-based ELISA using TZM-bl cells expressing CD4 and rabbit anti-HIV-1 gp120 antibody. (C) The binding between DLDs and N63 peptide derived from gp41 NHR was measured with ELISA using mouse anti-His-Tag monoclonal antibody. (D) The binding between DLDs and N63-trimer was measured with ELISA using mouse anti-His-Tag monoclonal antibody. (E) Measurement of inhibitory activity of DLDs on 6-HB formation between N36 and C34-FAM peptides by FN-PAGE. DLDs were incubated with N36 peptide at 37°C for 30 min before addition of C34-FAM. After incubation for 30 min, the mixtures were analyzed by FN-PAGE using an imaging system through U.V. detection (panels a and b). After imaging, the gels were stained with Coomassie blue (panels c and d).

To evaluate the antiviral mechanism of DLDs, we measured their binding affinity to gp120 and gp41. Through ELISA, we found that the DLDs bound gp120 with an affinity similar to that of mD1.22 binding to gp120, while D5 scFv showed no apparent binding with gp120 (Fig. 3A). We then determined whether DLDs could block gp120 binding to the CD4 receptor on TZM-bl cells that stably expressed CD4, CCR5, and CXCR4 (28) using a cell-based ELISA. As shown in Fig. 3B, DLDs efficiently inhibited the binding of gp120 to CD4 as strongly as mD1.22, but, again, D5 scFv was unable to block gp120-CD4 binding. Meanwhile, we used N63 peptide derived from gp41 NHR and N63-trimeric protein to determine the binding effect of DLDs. The results showed that DLDs bind with N63 peptide and N63-trimer as strongly as D5 scFv, while mD1.22 not (Fig. 3C and D). Because 6-HB formation is critical during HIV-1 Env-mediated membrane fusion, we further conducted a fluorescence native polyacrylamide gel (FN-PAGE) assay (29) to detect whether DLDs could block 6-HB formation like D5 scFv. N36 peptide and FAM-labeled C34 peptide at identical concentrations were mixed to mimic the 6-HB core formation (29). When the mixture of N36 peptide and FAM-C34 peptide at equal concentration was run in the gel, we saw two fluorescence bands, the FAM-C34 band and the fluorescence 6-HB band in the lower and middle positions in the gel, respectively (Fig. 3Ea and 3Ec). No bands were revealed in FN-PAGE if only N36 peptide, DLDs, mD1.22, and D5 scFv were run in the gels because they were not labeled with FAM (Fig. 3Ea) and they carried net positive charges (Fig. 3Ec). When DLDs were mixed with FAM-C34 peptide before adding N36 peptide, we saw the C34 bands, but no 6-HB bands were observed (Fig. 3Ea and 3Ec), suggesting that DLDs can effectively block 6-HB formation between N36 and FAM-C34 peptides (Fig. 3Ea and Ec). As a positive control, D5 scFv also inhibited 6-HB formation between N36 and FAM-C34 peptides, while the negative-control mD1.22 could not (Fig. 3Eb and 3Ed). Thus, through their D5 scFv component, DLDs were now able to interact with gp41 NHR, resulting in the inhibition of 6-HB formation between N36 and FAM-C34 peptides.

### Dual-targeting recombinant protein DLDs with 35-mer linker exhibits the best activity against HIV-1 infection.

**FIG 4:**
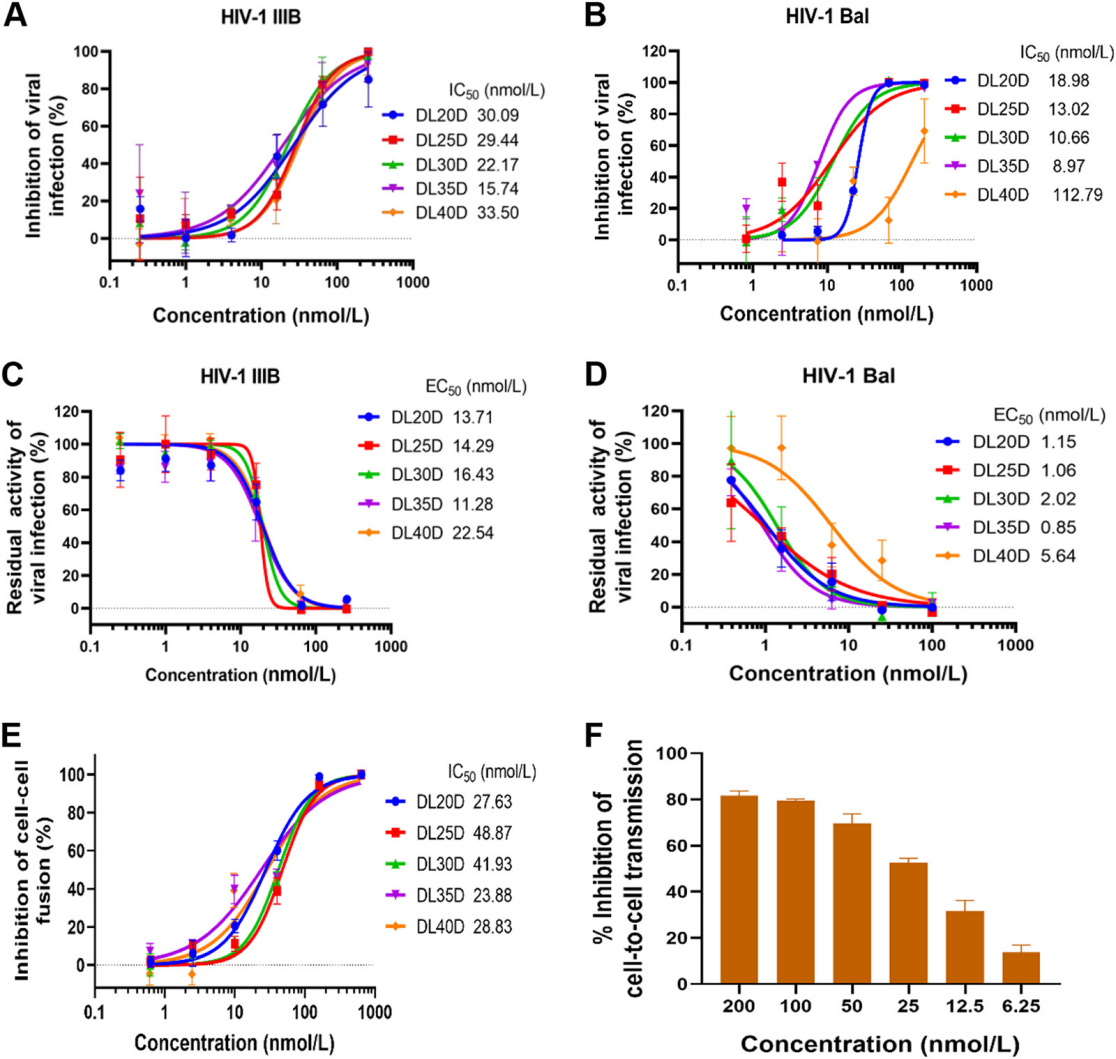
Anti-HIV-1 activity of DLDs *in vitro*. (A-B) Inhibitory activity of DLDs against infection by HIV-1 laboratory-adapted strains IIIB (X4) and Bal (R5). MT-2 and M7 cells were used for infection by HIV-1 IIIB and Bal, respectively. (C-D) Inactivation activity of DLDs against cell-free HIV-1 IIIB and Bal virions. (E) Inhibitory activity of DLDs on HIV-1 Env-mediated cell-cell fusion. The HIV-1_IIIB_ chronically infected H9 (H9/HIV-1_IIIB_) cells and CD4^+^ MT-2 cells were used as the effector and target cells in the cell-cell fusion assay. (F) Inhibitory activity of DL35D on HIV-1 cell-to-cell transmission.

We next evaluated the inhibitory and inactivation activity of the above five DLDs against divergent HIV-1 strains, hoping to select a DLD with the highest anti-HIV-1 activity for subsequent study. As shown in Fig. 4A and B, all five DLDs could inhibit infection of the HIV-1 laboratory-adapted strains IIIB and Bal with IC_50_ values in the range of 15.7 to 33.5 and 9.0 to 112.8 nmol/liter, respectively. Moreover, DLDs efficiently inactivated cell-free HIV-1 IIIB and Bal virions with EC_50_ values in the range of 11.28 to 22.54 and 0.85 to 5.64 nmol/liter, respectively (Fig. 4C and D). These DLDs also effectively inhibited HIV-1 Env-mediated cell-cell fusion, as determined using a cell-cell fusion assay as previously reported (30), with EC_50_ values ranging from 23.88 to 48.87 nmol/liter (Fig. 4E). DL35D, the recombinant protein containing a 35-mer linker, showed the best HIV-1 inhibitory and inactivation activity, as well as cell-cell fusion inhibitory activity, among all five DLDs. These results suggest that (GGGGS)_7_ is the linker with optimal length for the construction of recombinant bifunctional proteins, consistent with our previous observation (31). A previous report demonstrated that cell-to-cell transmission of HIV-1 is more efficient than the release of cell-free virions (32). We also evaluated the inhibitory activity of DL35D against HIV-1 cell-to-cell transmission as previously reported (33). As shown in Fig. 4F, DL35D effectively inhibited HIV-1 cell-to-cell transmission with 53% inhibition at the concentration of 25 nmol/liter.

Then, we tested the inhibitory activity of DL35D against infection by HIV-1 primary isolates with different subtypes and tropism as well as T20-resistant strains. We found that DL35D potently inhibited infection of divergent HIV-1 primary isolates, including 92UG029 (A, X4), 93BR020 (F, X4/R5), BCF02 (O, X4), 92TH009 (CRF01_AE, R5), and 92UG024 (D, X4), with IC_50_ values ranging from 3 to 17.9 nmol/liter, and suppressed the infection of T20-resistant strains with IC_50_ values ranging from 9.3 to 16 nmol/liter (Table 1). More interestingly, DL35D effectively inactivated cell-free HIV-1 virions with EC_50_ of 0.6 ∼ 5 nmol/liter (Table 2). These results suggest that DL35D is a promising candidate for development as a novel HIV-1 entry inhibitor and HIV-1 inactivator for treatment and prevention of HIV-1 infection.

**TABLE 1:** Inhibitory activity of DL35D against HIV-1 primary isolates and T20-resistant strains (IC_50_)

Virus isolates	mD1.22 (nmol/L)	D5 scFv (nmol/L)	DL35D (nmol/L)
Primary HIV-1 isolates			
92UG029 (A, X4)	3.22 ± 0.62	>250	3.02 ± 0.35
93BR020 (F, X4/R5)	18.23 ± 5.40	>250	16.95 ± 5.92
BCF02 (O, X4)	24.96 ± 0.73	>250	17.92 ± 4.58
92TH009 (CRF01_AE, R5)	11.86 ± 2.95	>250	6.69 ± 1.30
92UG024 (D, X4)	4.04 ± 1.61	>250	4.66 ± 0.16
T20-resistant virus (HIV-1_NL4-3_ [36G])			
(D36G) V38A/N42D	15.83 ± 1.25	>250	15.98 ± 4.01
(D36G) N42T/N43K	13.89 ± 3.54	>250	11.05 ± 2.29
(D36G) V38E/N42S	13.02 ± 3.82	>250	9.27 ± 1.08

**TABLE 2:** Inactivation activity of DL35D against HIV-1 primary isolates and T20-resistant strains (EC_50_)

Virus isolates	mD1.22 (nmol/L)	D5 scFv (nmol/L)	DL35D (nmol/L)
Primary HIV-1 isolates			
92UG029 (A, X4)	5.46 ± 1.31	>250	5.22 ± 2.02
92UG024 (D, X4)	13.50 ± 4.46	>250	3.92 ± 1.28
93BR020 (F, X4/R5)	2.70 ± 0.75	>250	2.78 ± 1.02
T20-resistant virus (HIV-1_NL4-3_ [36G])			
Parental	1.30 ± 0.59	>250	1.03 ± 0.34
V38A	1.97 ± 0.47	>250	2.03 ± 1.05
V38A/N42D	1.45 ± 0.41	>250	0.60 ± 0.05

### Toxin-conjugated recombinant protein DL35D-DM1 efficiently inhibits HIV-1 infection.

In order to improve the inactivation activity of DL35D against cell-free HIV-1 virions and HIV-1-infected cells, we conjugated DL35D with a linker-toxin, SMCC-DM1, to construct a toxin-conjugated recombinant protein, DL35D-DM1 (Fig. S3). After the mD1.22 component in DL35D-DM1 binds to gp120, it is expected that the D5 scFv component in DL35D-DM1 will bind the exposed NHR-trimer and that the DM1 will be released to the target cell, causing apoptosis (Fig. 1Ac).

10.1128/mbio.03384-21.3 FIG S3 Analysis of the proteins DLD and toxin-conjugated protein by SDS-PAGE. Download FIG S3, PDF file, 0.04 MB. Copyright © 2022 Wang et al. 2022 Wang et al. https://creativecommons.org/licenses/by/4.0/ This content is distributed under the terms of the Creative Commons Attribution 4.0 International license.

**FIG 5:**
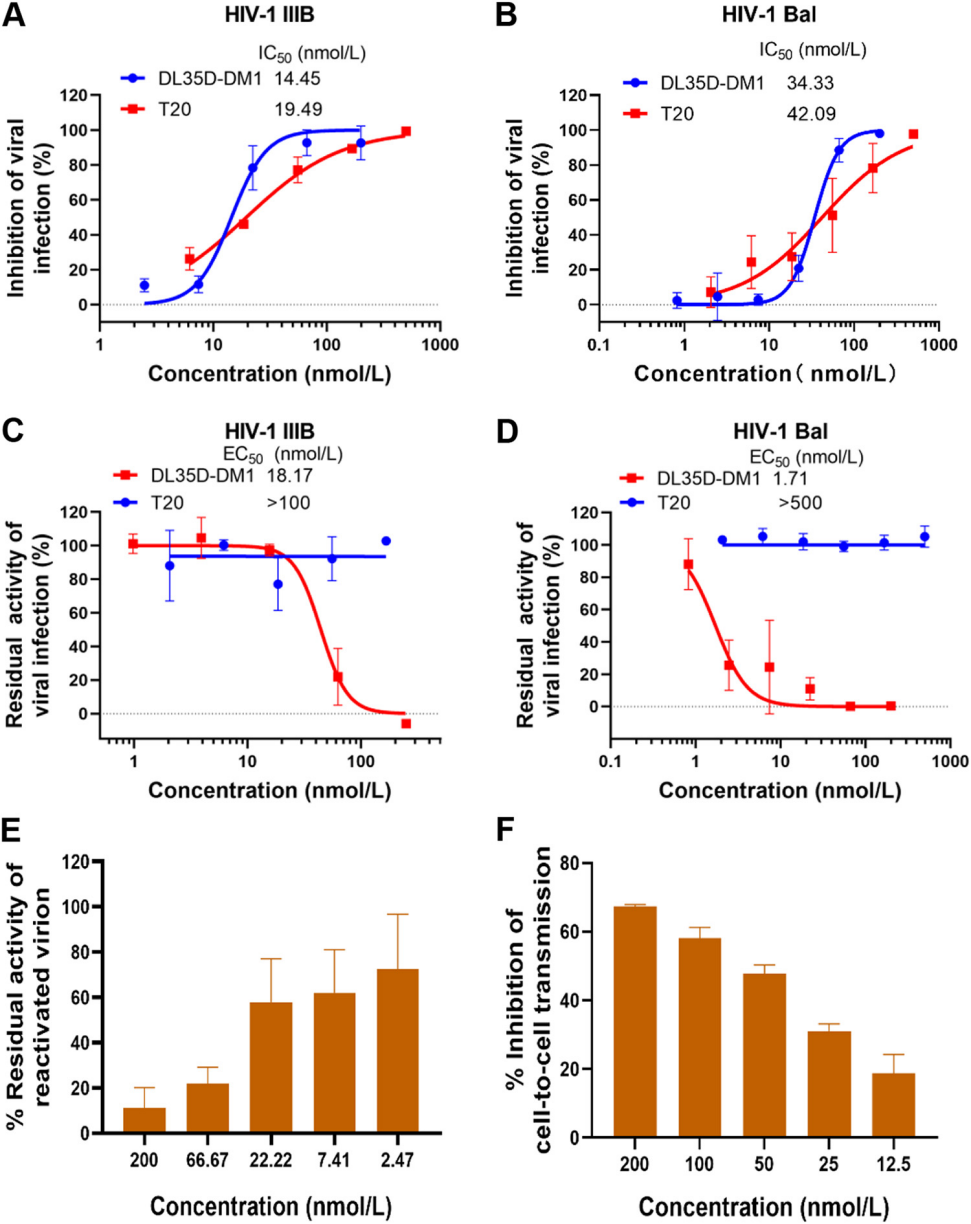
Activity of DL35D-DM1 against HIV-1 strains *in vitro*. (A-B) Inhibitory activity of DL35D-DM1 against infection by HIV-1 laboratory-adapted strains (IIIB and Bal). (C-D) Inactivation activity of DL35D-DM1 against cell-free HIV-1 particles (HIV-1 IIIB and Bal). (E) Inactivating LRA-reactivated HIV-1 virions released from ACH-2 cells by DL35D-DM1. (F) Inhibition of HIV-1 cell-to-cell transmission by DL35D-DM1.

To test our hypothesis, we first evaluated the inhibitory and inactivation activity against some HIV-1 strains. As shown in Fig. 5A and B, DL35D-DM1 inhibited infection of the HIV-1 laboratory-adapted strain IIIB and Bal with the IC_50_ values of 14.45 and 34.33 nmol/liter, respectively. Meanwhile, DL35D-DM1 efficiently inactivated HIV-1 IIIB and Bal virions with EC_50_ of 18.17 and 1.71 nmol/liter, respectively (Fig. 5C and D). Notably, DL35D-DM1 potently inactivated the virions of HIV-1 primary isolates, including 92UG029 (A, X4), 92UG024 (D, X4), 93BR020 (F, X4/R5), and 92TH009 (CRF01_AE, R5), with EC_50_ values in the range of 6.82 ∼ 15.4 nmol/liter (Table 3). Next, we evaluated the inactivation effect of DL35D-DM1 against HIV-1 virions released from romidepsin-reactivated ACH-2 cells. As shown in Fig. 5E, DL35D-DM1 at the concentration of about 20 nmol/liter inactivated more than 50% infectious viral particles. Like DL35D, DL35D-DM1 also inhibited HIV-1 cell-to-cell transmission in a dose-dependent manner (Fig. 5F). These results suggest that DL35D-DM1 exhibits potent inhibition and inactivation activity against cell-free HIV-1.

**TABLE 3:** Inhibitory and inactivation activity of DL35D-DM1 against HIV-1 primary isolates (EC_50_)

Primary isolates	Inactivation activity (nmol/L)
92UG029 (A, X4)	15.40
92UG024 (D, X4)	11.30
93BR020 (F, X4/R5)	6.82
92TH009 (CRF01_AE, R5）	8.85

### Toxin-conjugated recombinant protein DL35D-DM1 specifically inactivates HIV-1-infected cells and LRA-reactivated latent cells.

**FIG 6:**
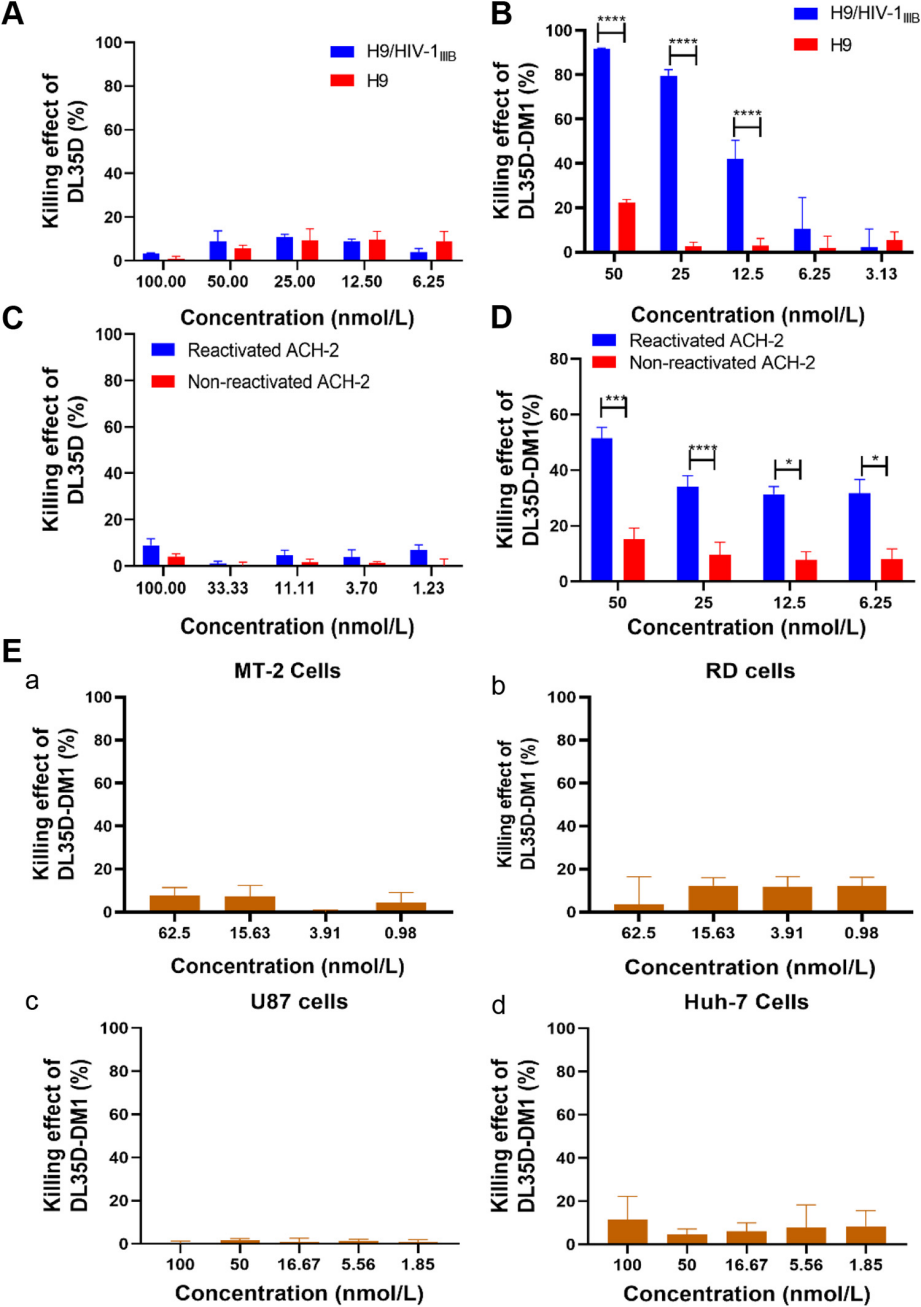
Killing effect of DL35D-DM1 on HIV-1-infected cells. (A) Killing effect of protein DL35D on HIV-1-infected H9/HIV-1_IIIB_ cells and uninfected H9 cells. (B) Killing effect of DL35D-DM1 on H9/HIV-1_IIIB_ cells and H9 cells. (C) Killing effect of protein DL35D on LRA-reactivated HIV-1 latent-infected ACH-2 cells. (D) Killing effect of DL35D-DM1 on LRA-reactivated HIV-1 latent-infected ACH-2 cells. Nonreactivated ACH-2 cells were used as control. (E) Killing effect of DL35D-DM1 on cells without expression of HIV-1 Env. MT-2 cells (a), RD cells (b), U87 CD4+CCR5+ cells (c), and Huh-7 cells (d) were used in this experiment. Two-way ANOVA was used in the statistical analysis. ****, ***, and * mean *P* < 0.0001, *P* < 0.001, and *P* < 0.05, respectively.

To determine whether DL35D-DM1 can specifically kill HIV-1-infected cells, we evaluated its killing effect on HIV-1 IIIB chronically infected H9 (H9/HIV-1_IIIB_) cells and romidepsin-reactivated ACH-2 cells. As shown in Fig. 6A, DL35D had no significant killing effect on H9/HIV-1_IIIB_ cells and H9 cells at the concentration up to 100 nmol/liter (Fig. 6A). In contrast, DL35D-DM1 showed potent killing effect on H9/HIV-1_IIIB_ cells in a dose-dependent manner, with an EC_50_ of 14.48 nmol/liter (Fig. 6B). For the negative-control H9 cells, DL35D-DM1 exhibited only 22% killing effect at a concentration of 50 nmol/liter. Then, we tested the killing effect of DL35D-DM1 on ACH-2 cells stimulated with romidepsin, an LRA, for 12 h at the concentration of 5 nmol/liter. Similarly, DL35D had no apparent killing effect on either LRA-activated or nonactivated ACH-2 cells at 100 nmol/liter (Fig. 6C), while DL35D-DM1 at 50 nmol/liter could kill 51% and 15% of LRA-reactivated and nonreactivated ACH-2 cells, respectively (Fig. 6D). Finally, we evaluated the killing effect of DL35D-DM1 on other human cell lines as controls, including a human T cell line (MT-2), a human rhabdomyosarcoma cell line (RD), a human hepatocyte cell line (Huh-7), and a human glioblastoma cell line (U87 CD4+CCR5+), which are derived from primary cells in different human tissues. We found no significant killing effect of DL35D-DM1 at the indicated concentrations (Fig. 6E). These results demonstrated that this toxin-conjugated dual-targeting recombinant protein DL35D-DM1 could specifically and efficiently kill HIV-1-infected cells and LRA-reactivated ACH-2 cells, suggesting its superior ability to eliminate HIV-1-infected cells in the blood and organs, and the LRA-reactivated latently infected cells in the HIV latent reservoir.

## DISCUSSION

Even with increasing access to the highly effective cART, HIV-1 still threatens the world's public health, thus far claiming 36.3 million lives (https://www.who.int/news-room/fact-sheets/detail/hiv-aids). Nevertheless, along with the successful control of HIV-1 replication by antiretroviral drugs, HIV-1 infection has become a manageable chronic health condition, even though lacking a cure for HIV-1/AIDS. The long-lived HIV reservoir, including latently infected resting memory CD4^+^ T cells, is considered one of the major obstacles to an HIV/AIDS cure (2, 3, 34). The persistence of HIV-1 makes it necessary for patients to comply with long-term medication regimens, affecting the quality of life and risking the emergence of drug resistance. To overcome this obstacle, several research groups have proposed the “shock and kill” strategy, i.e., using LRAs to reactivate HIV latent cells and then killing the newly produced virions and HIV-infected cells by antiviral drugs or host immune system in order to eradicate the latent HIV reservoirs (5, 35). Many LRAs are highly effective in reactivating HIV latent cells, such as HDACi (36). Romidepsin is one of the most efficient HDACis (37). We chose romidepsin as an LRA to reactivate ACH-2 cells because it was reported that romidepsin could effectively reactivate the HIV reservoir and reduced the reservoir size when combined with a therapeutic vaccine in a clinical trial (38).

Although cART is highly effective in the inhibition of HIV replication, it cannot kill HIV and HIV-infected cells. So far, very few antiviral drugs with capacity to kill cell-free HIV virions and HIV-infected cells have been reported. Some antibody-drug conjugates (ADCs), in which HIV-1 Env-specific antibodies are conjugated with toxin, have shown specific killing effect *in vitro* and *in vivo* (33, 39, 40), but they have difficulty in accessing the tissues with HIV latent reservoirs because of their large molecular size. It has been shown that an immunotoxin targeting the CD4-binding site in gp120, CD4-PE40, could specifically kill HIV-1-infected cells (11). However, the low efficiency of CD4-PE40 and the high immunogenicity against PE40 in clinical trials have limited its further development (18). Different from the toxic protein (PE40) in CD4-PE40, the toxic molecule in DL35D-DM1, DM1, is not a protein, but a small-molecule compound, which is expected to have no or low immunogenicity to humans in clinical trials in the future.

In this study, we aimed to design and characterize a toxin-conjugated recombinant protein with lower molecular size and higher killing activity against HIV-1 and HIV-1-infected cells or LRA-reactivated HIV latent cells. We first constructed a CD4-PE40-like recombinant protein by using mD1.22 to replace CD4 and DM1 to substitute for PE40 because 1) mD1.22 has more potent anti-HIV-1 activity (20) and is safer than sCD4, which could enhance HIV-1 infection in CD4-CCR5^+^ cells (13, 18), and 2) a recombinant protein with PE40 (MW: 66 kDa) may have less accessibility to the limited space of HIV latent reservoir than that containing the small-molecule toxin DM1. Interestingly, mD1.22-DM1 was able to bind to gp120, inhibit HIV-1 infection, inactivate cell-free HIV-1 virions, but could not significantly kill LRA-activated latent cells (Fig. 1), possibly because the ACH-2 cells is not fully reactivated at low concentration of LRA (16). In addition, the rapid shedding of gp120, after its binding with a CD4 molecule, from HIV-1-infected cells or LRA-reactivated latent cells may further reduce the cell-killing effect of mD1.22-DM1. These results suggest that the specific killing effect of a toxin-conjugated protein targeting only CD4 binding site in gp120 might be too weak to kill LRA-reactivated latent cells. Therefore, we decided to construct a recombinant protein with multiple targets in order to improve its capacity to kill HIV-1-infected cells and LRA-reactivated latent cells.

Compared with a single-target drug, a multitarget drug generally has more advantages, including higher binding affinity, better efficiency, and better functionality because of the potential synergistic effect of acting on different targets (41). We previously constructed the dual-targeting protein-based HIV-1 inactivator 2DLT, which is comprised of the D1D2 domains of CD4 that target the CD4-binding site in gp120 and a peptide-based HIV-1 fusion inhibitor targeting the NHR domain in gp41. We reported that it was more efficient in inhibiting HIV-1 infection and inactivating cell-free HIV-1 virions than D1D2 alone. Unlike sCD4 or D1D2, 2DLT did not enhance HIV-1 infection in CD4-/CCR5^+^ cells, making it safer to use (29). Protein-based viral inactivators are recombinant or nonrecombinant proteins that can attack the cell-free virion directly through interaction with viral envelop protein, inducing its conformational change or destroying the integrity of the viral membrane, resulting in the loose of its infectivity (42).

**FIG 7:**
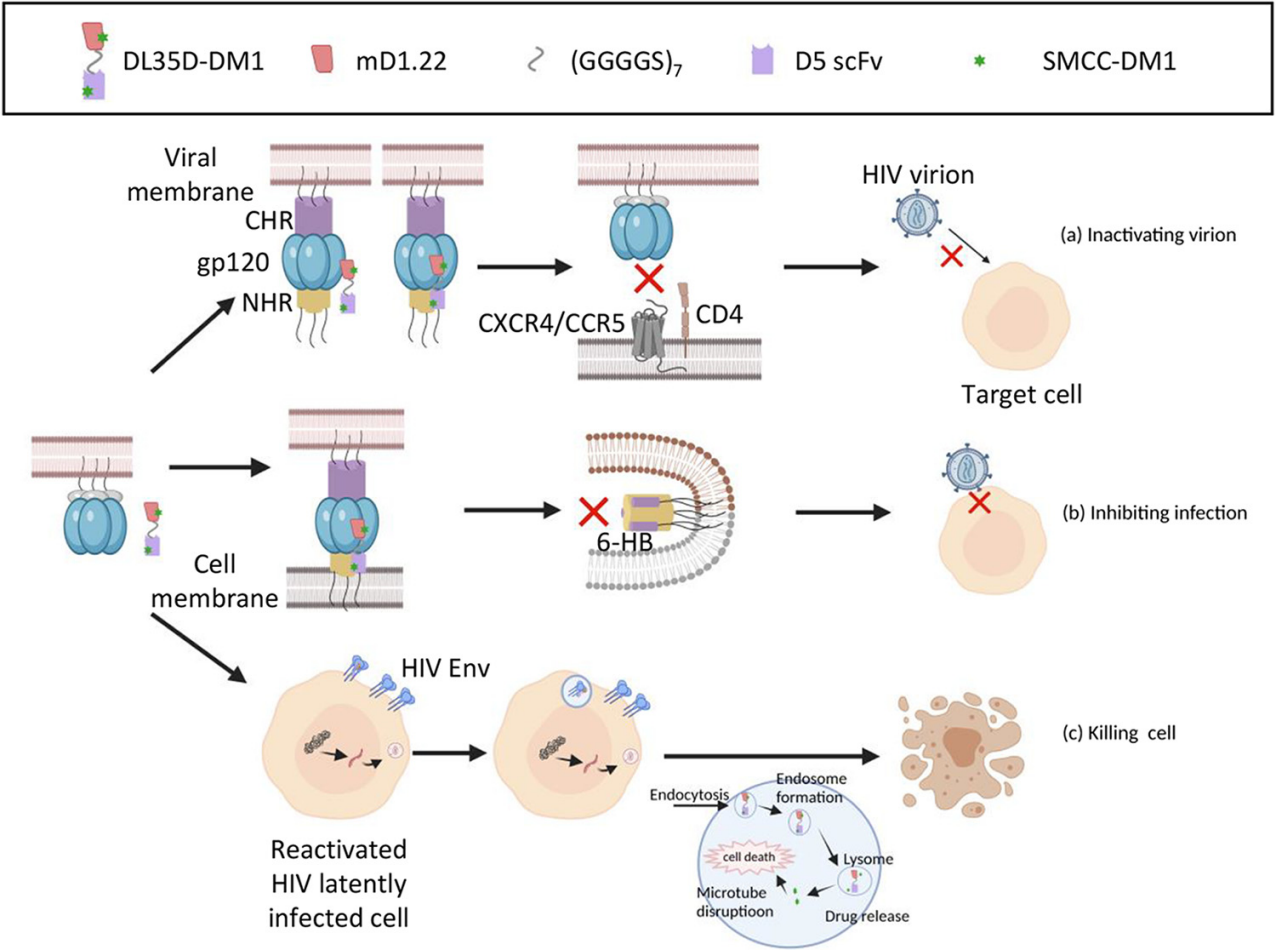
The putative mechanisms of action of DL35D-DM1 to inhibit HIV-1 infection, inactivate cell-free HIV-1 virions and kill cells expressing HIV-1 Env. (a) The mechanism of DL35D-DM1 to inactivate cell-free HIV-1 virions. DL35D-DM1 binds HIV-1 gp120 to trigger its conformational change and then binds to exposed gp41 NHR trimer, resulting in the inactivation of HIV-1 virion. (b) The mechanism of DL35D-DM1 to inhibit HIV-1 infection. DL35D-DM1 binds to gp120 to block the interaction between gp120 and cell receptors or binds to gp41 to block 6-HB formation, resulting in inhibition of HIV-1 infection. (c) The mechanism of DL35D-DM1 to kill LRA-reactivated HIV-1 latently infected cell or HIV-1-infected cell. DL35D-DM1 binds to HIV-1 Env expressed on the surface of cells (e.g., LRA-reactivated HIV-1 latently infected cell or HIV-1-infected cell) and gets into the endosome in the cell, where the released DM1 to disrupt the microtube system, resulting cell death. The figure was created with BioRender.com.

The exact distance between the CD4-binding site in gp120 and the NHR domain in gp41 is unknown. Therefore, we constructed five dual-targeting recombinant proteins, DLDs, by linking mD1.22 and D5 scFv expected to bind the CD4-binding site in gp120 and the NHR domain in gp41, respectively, with different length of flexible linkers (GGGGS)_n_. All five DLDs showed efficient inhibition and inactivation activity against HIV-1 laboratory-adapted strains. However, DL35D, which contains a 35-mer linker, bound to both gp120 and gp41, had the highest inhibitory activity against infection by divergent HIV-1 strains, including those resistant to T20. It blocked HIV-1-mediated cell-cell fusion and HIV-1 cell-to-cell transmission, as well as inactivate cell-free HIV-1 particles, suggesting that the flexible 35-mer linker is sufficient to allow the mD1.22 and D5 scFv parts in DL35D to interact simultaneously with the CD4-binding site in gp120 and NHR domain in gp41, respectively. We then conjugated DM1 to DL35D using a method to generate mD1.22-DM1 similar to that described above. As expected, DL35D-DM1 efficiently inactivated cell-free HIV-1 virions and kill HIV-1 chronically infected H9/HIV-1_IIIB_ cells, as well as romidepsin-reactivated latent ACH-2 cells, in a dose-dependent manner, while it had no significant killing effect on H9 cells, nonreactivated ACH-2 cells, and other CD4^+^ cells because these cells do not express HIV-1 gp120 and gp41. These results confirm that DL35D-DM1, targeting both CD4-binding site in gp120 and NHR domain in gp41, is much more potent in killing LRA-reactivated latent cells than mD1.22-DM1 that only targets the CD4-binding site in gp120, providing theoretical and experimental evidence for guiding the rational design of toxin-conjugated multitarget or multifunctional viral inactivators in the future. The putative mechanisms of action of DL35D-DM1 are illustrated in Fig. 7. First, DL35D-DM1 binds to HIV-1 gp120 through its mD1.22 part to trigger a conformational change of gp120 and the exposure of gp41 NHR trimer, to which DL35D-DM1 further binds through its D5 scFv part, resulting in the inactivation of HIV-1 virion (Fig. 7a). Second, during HIV-1 infection, DL35D-DM1 binds to gp120 through its mD1.22 part, blocking the interaction between gp120 and cellular receptor. DL35D-DM1 could also bind to gp41 NHR-trimer through its D5 scFv part, blocking the 6-HB formation. Either of the above actions could inhibit HIV-1 infection (Fig. 7b). Third, DL35D-DM1 binds to HIV-1 Env on the cell surface and enters into the cell through endocytosis. Then, DL35D-DM1 is degraded in the lysosome to yield metabolites containing DM1 with linker SMCC and lysine (23), where DM1 is able to disrupt the microtube system and induce cell death (Fig. 7c).

The only limitation of this approach is that a toxin-conjugated antiviral protein may have nonspecific killing effect on noninfected cells at high concentration. For example, DL35D-DM1 at 50 nmol/liter could kill 22% and 15% of H9 and nonreactivated ACH-2 cells, respectively. Therefore, it is necessary to further optimize the ratio of toxin and protein to be conjugated, select a better linker with higher resistance to proteolytic enzymes, and improve coupling efficiency in order to reduce the off-target effect. Study on the effect of DL35D-DM1 treatment on the eradication of HIV-1 latent reservoir in an animal model is recommended.

## MATERIALS AND METHODS

### Cells and viruses.

MT-2 cells, CEMx174 5.25 M7 cells, ACH-2 cells, H9 cells, and H9/HIV-1_IIIB_ cells were obtained from the NIH AIDS Reagent Program and cultured with RPMI 1640 medium containing 10% fetal bovine serum (FBS). Huh-7 cells, RD cells, U87 CD4+CCR5+ cells and TZM-bl cells were stocked in our lab and cultivated in DMEM medium with 10% FBS. Expi293 cells were cultured with the SMM 293-TII Expression Medium (Sino Biological, China). Viruses used in this study were also obtained from the NIH AIDS Reagent Program.

### Expression of proteins and construction of toxin-conjugated recombinant protein.

The recombinant protein mD1.22 was expressed and purified as described previously. According to a previous report, the tubulin inhibitor DM1 containing linker SMCC was purchased from MedChemExpress (Cat. No.: HY-101070, Molecule weight: 1072.61 Da) and coupled with antibodies or proteins by lysine reaction (43–45). Briefly, the protein was mixed with SMCC-DM1 at the molar ratio of 1:5 and reacted for 4 h at room temperature. Then the reaction mixture was centrifuged with Amicon Ultra to remove the unconjugated SMCC-DM1 and organic solvent. Finally, the concentration of toxin-conjugated protein was determined by Nanodrop spectrophotometer and TaKaRa BCA Protein assay kit.

According to the previous report (29, 31), we selected flexible amino acid linker (GGGGS)_n_ with different lengths to link mD1.22 and D5 scFv. The genes encoding recombinant protein mD1.22-linker-D5 scFv (DLDs) were synthesized by Shanghai HuaGen Biotech Co., Ltd. and digested by restriction enzymes SfiI and XhoI, followed by construction of the expression vector pSecTag2B. Their sequences were confirmed by sequencing subsequently. As in the previous study, the proteins were expressed in Expi293 cells and purified with Ni Smart beads (22). The proteins were then analyzed with rabbit anti-human CD4 polyclonal antibody (Proteintech, China), mouse anti-mD1.22, and mouse anti-D5 scFv serum in ELISA and Western blot. Mouse anti-mD1.22 and D5 scFv serum were obtained in our lab through immunizing a mouse with the adjuvant Alum. For Western blot, purified proteins were mixed with loading buffer, respectively, and boiled for 5 min. Electrophoresis was conducted in SDS running buffer at 120V for 1 h. Then the proteins were transferred to the PVDF membrane at 200 mA for 30 min.

### ELISA and cell-based ELISA.

For detection of the binding effect between proteins and gp120, gp120 was coated onto a 96-well polystyrene plate at 10 μg/ml at 4°C overnight. Then, the plate was blocked with 2% nonfat milk at 37°C for 2 h, followed by the addition of recombinant protein DLDs. Similarly, to determine the binding effect between fusion proteins and gp41, the N63 peptide derived from gp41 and N63-trimeric protein were coated at 5 μg/ml, respectively, followed by adding recombinant proteins DLDs. Mouse anti-His-Tag monoclonal antibody (Proteintech, China) was used to determine the binding effect.

Cell-based ELISA was conducted to measure whether DLDs could block gp120 binding to the CD4 receptor. Briefly, TZM-bl cells expressing CD4 were seeded into a 96-well plate at 2 × 10^5^ cells/ml and cultured overnight. Then the cells were blocked with blocking buffer (PBS with 2% nonfat milk and 3% BSA) at 37°C for 2 h. The protein DLDs (10 μM) and gp120 (5 μg/ml) were mixed and added into TZM-bl cells. After incubation at 37°C for 1 h, HIV-1 gp120 antibody (Sino Biological, China) was added and allowed to incubate for another 1 h. The effect was determined by HRP-conjugated Goat anti-Rabbit antibody (Dako, Denmark).

### Inactivation of HIV-1 strains.

Recombinant protein-mediated HIV-1 inactivation activity was determined as previously described (29). Briefly, 100 μl diluted recombinant protein were mixed with HIV-1 at 500 TCID_50_ (50% tissue culture infectivity dose) and incubated at 4°C for 1 h. Next, PEG-6000 was added at a final concentration of 3% to precipitate the virus at 4°C for 1 h. After that, the protein-virus mixture was centrifuged at 15,000 rpm for 30 min and washed 3x with 3% PEG containing 10 mg/ml BSA. The virus pellet was resuspended with RPMI 1640 and cultured with MT-2 cells (X4 virus) or TZM-bl cells (R5 virus). For the X4 virus, after a 5-day infection, the supernatants were collected and tested for p24 expression level with ELISA. For R5 virus, luciferase was detected 2 days postinfection as described previously (22). The median effective concentration (EC_50_) was calculated by using the CalcuSyn program as previously described (22).

### Inhibition of HIV-1 infection.

The inhibition assay was determined as previously described (30). Briefly, HIV-1 at 100 TCID_50_ was incubated with diluted proteins at 37°C for 30 min. Following this, 1 × 10^4^ MT-2 cells (X4 virus), M7 cells, or TZM-bl cells (X4 or R5 cells) per well were counted and added to the virus-protein mixture. After 10 h of infection, the supernatants were replaced with fresh medium containing 10% FBS. For MT-2 cells or M7 cells, the supernatant was collected after an additional 5-day culture and treated with 5% Triton X-100. The expression level of p24 was detected by ELISA as previously described (29). For TZM-bl cells, luciferase was detected 2 days postinfection and IC_50_ was calculated by using the CalcuSyn program as previously described (22).

### Inhibition of HIV-1 Env-mediated cell-cell fusion.

H9/HIV-1_IIIB_ cells were labeled with calcein-AM at 37°C for 30 min and washed with PBS twice. Then the cells were mixed with graded dilution proteins and cultured at 37°C for 30 min. After incubation, MT-2 cells were added and cultured for an additional 2 h. Fusion cells were observed under a fluorescence microscope. Five photos were taken for each well. The fused and unfused cells were then counted. The inhibition rate of HIV-1 Env-mediated fusion cells and the IC_50_ values were calculated as described previously (29).

### Inhibition of HIV-1 cell-to-cell transmission.

The activities of recombinant proteins in inhibiting HIV-1 cell-to-cell transmission were tested as described previously (33). Briefly, TZM-bl cells were seeded into a 96-well plate at 1 × 10^5^/ml and cultivated overnight. The recombinant protein was diluted with DMEM medium and transferred to TZM-bl cells. Then 6,000 H9/HIV-1_IIIB_ cells were added to the TZM-bl cells. After co-incubation for 6 h at 37°C, the supernatants containing protein and cells were removed, and the TZM-bl cells were washed with PBS three times. Luciferase was detected 48 h later and % inhibition of HIV-1 cell-to-cell transmission was calculated as previously described (33).

### Fluorescence native polyacrylamide gel electrophoresis (FN-PAGE).

FN-PAGE used to detect 6-HB formation was performed according to the previous report (29). Briefly, the fusion protein (30 μM) was pre-incubated with N63 peptide (100 μM) at 37°C for 30 min, and then the C34 peptide (100 μM) was added to the mixture and incubated for an additional 30 min. Afterwards, the samples were mixed with a high-pH loading buffer and analyzed by native gel electrophoresis at 120 V for 2 h on ice. The gels were visualized with the Gel Image System (Tanon 1600) and stained with Coomassie blue.

### Reactivation of ACH-2 cells.

HIV-1 latent-infected ACH-2 cells were seeded into a six-well plate at the concentration of 1 × 10^5^ cells/ml, followed by the addition of romidepsin to cells with different concentrations. After 12 h of stimulation, the cells and supernatants were collected to detect Env-expression level on the cell surface by flow cytometry and p24 level on the supernatants by ELISA. Briefly, ACH-2 cells were washed with PBS and incubated with N6 antibody (46) at a final concentration of 20 μg/ml on ice for 1 h. Then the cells were washed with PBS three times and incubated with Goat anti-human IgG (FITC) on ice for 45 min. After that, the cells were washed with PBS four times and tested with flow cytometry. ELISA was conducted as above.

### Cell-killing effect.

HIV-1-infected H9/HIV-1_IIIB_ cells and reactivated latently infected ACH-2 cells were used to determine the cell-killing effect of toxin-conjugated recombinant protein; uninfected H9 cells and nonreactivated ACH-2 cells were used as control. The toxin-conjugated recombinant protein mD1.22-DM1 or DL35D-DM1 was diluted with DMEM, respectively, followed by the addition of cells (1 × 10^4^) and incubation for 3 days. The killing effect on cells was detected using CCK8 kit (DOJINDO, Japan) (47). Latent-infected ACH-2 cells were activated with the LRA, romidepsin.

### Statistical analysis.

Analysis of Variance (ANOVA) was used in the statistical analysis of data between groups *in vitro*. *P* < 0.05 indicates statistical difference. **** means *P* < 0.0001, *** means *P* < 0.001, ** means *P* < 0.01, and * means *P* < 0.05, NS means no statistical significance.

## References

[1] Barré-Sinoussi F, Chermann JC, Rey F, Nugeyre MT, Chamaret S, Gruest J, Dauguet C, Axler-Blin C, Vézinet-Brun F, Rouzioux C, Rozenbaum W, Montagnier L. 1983. Isolation of a T-Lymphotropic Retrovirus From A Patient At Risk For Acquired Immune-Deficiency Syndrome (AIDS). Science 220:868–871. doi:10.1126/science.6189183. 6189183

[2] Deeks SG, Overbaugh J, Phillips A, Buchbinder S. 2015. HIV infection. Nat Rev Dis Primers 1:15035. doi:10.1038/nrdp.2015.35. 27188527

[3] Finzi D, Hermankova M, Pierson T, Carruth LM, Buck C, Chaisson RE, Quinn TC, Chadwick K, Margolick J, Brookmeyer R, Gallant J, Markowitz M, Ho DD, Richman DD, Siliciano RF. 1997. Identification of a reservoir for HIV-1 in patients on highly active antiretroviral therapy. Science 278:1295–1300. doi:10.1126/science.278.5341.1295. 9360927

[4] Xu W, Li H, Wang Q, Hua C, Zhang H, Li W, Jiang S, Lu L. 2017. Advancements in developing strategies for sterilizing and functional HIV cures. Biomed Res Int 2017:6096134. doi:10.1155/2017/6096134. 28529952 PMC5424177

[5] Deeks SG. 2012. HIV: shock and kill. Nature 487:439–440. doi:10.1038/487439a. 22836995

[6] Thorlund K, Horwitz MS, Fife BT, Lester R, Cameron DW. 2017. Landscape review of current HIV “kick and kill” cure research - some kicking, not enough killing. BMC Infect Dis 17:595. doi:10.1186/s12879-017-2683-3. 28851294 PMC5576299

[7] Siliciano JD, Kajdas J, Finzi D, Quinn TC, Chadwick K, Margolick JB, Kovacs C, Gange SJ, Siliciano RF. 2003. Long-term follow-up studies confirm the stability of the latent reservoir for HIV-1 in resting CD4(+) T cells. Nat Med 9:727–728. doi:10.1038/nm880. 12754504

[8] Weissenhorn W, Dessen A, Harrison SC, Skehel JJ, Wiley DC. 1997. Atomic structure of the ectodomain from HIV-1 gp41. Nature 387:426–430. doi:10.1038/387426a0. 9163431

[9] Lu M, Kim PS. 1997. A trimeric structural subdomain of the HIV-1 transmembrane glycoprotein. J Biomol Struct Dyn 15:465–471. doi:10.1080/07391102.1997.10508958. 9439994

[10] Chan DC, Fass D, Berger JM, Kim PS. 1997. Core structure of gp41 from the HIV envelope glycoprotein. Cell 89:263–273. doi:10.1016/S0092-8674(00)80205-6. 9108481

[11] Chaudhary VK, Mizukami T, Fuerst TR, FitzGerald DJ, Moss B, Pastan I, Berger EA. 1988. Selective killing of HIV-infected cells by recombinant human CD4-Pseudomonas exotoxin hybrid protein. Nature 335:369–372. doi:10.1038/335369a0. 2843774

[12] Berger EA, Clouse KA, Chaudhary VK, Chakrabarti S, FitzGerald DJ, Pastan I, Moss B. 1989. CD4-Pseudomonas exotoxin hybrid protein blocks the spread of human immunodeficiency virus infection in vitro and is active against cells expressing the envelope glycoproteins from diverse primate immunodeficiency retroviruses. Proc Natl Acad Sci USA 86:9539–9543. doi:10.1073/pnas.86.23.9539. 2480605 PMC298532

[13] Haim H, Si Z, Madani N, Wang L, Courter JR, Princiotto A, Kassa A, DeGrace M, McGee-Estrada K, Mefford M, Gabuzda D, Smith AB, 3rd, Sodroski J. 2009. Soluble CD4 and CD4-mimetic compounds inhibit HIV-1 infection by induction of a short-lived activated state. PLoS Pathog 5:e1000360. doi:10.1371/journal.ppat.1000360. 19343205 PMC2655723

[14] Clouse KA, Powell D, Washington I, Poli G, Strebel K, Farrar W, Barstad P, Kovacs J, Fauci AS, Folks TM. 1989. Monokine regulation of human immunodeficiency virus-1 expression in a chronically infected human T cell clone. J Immunol 142:431–438. 2463307

[15] Duh EJ, Maury WJ, Folks TM, Fauci AS, Rabson AB. 1989. Tumor necrosis factor alpha activates human immunodeficiency virus type 1 through induction of nuclear factor binding to the NF-kappa B sites in the long terminal repeat. Proc Natl Acad Sci USA 86:5974–5978. doi:10.1073/pnas.86.15.5974. 2762307 PMC297754

[16] Lan J, Yang K, Byrd D, Hu N, Amet T, Shepherd N, Desai M, Gao J, Gupta S, Sun Y, Yu Q. 2014. Provirus activation plus CD59 blockage triggers antibody-dependent complement-mediated lysis of latently HIV-1-infected cells. J Immunol 193:3577–3589. doi:10.4049/jimmunol.1303030. 25149467 PMC4170029

[17] Ramachandran RV, Katzenstein DA, Wood R, Batts DH, Merigan TC. 1994. Failure of short-term CD4-PE40 infusions to reduce virus load in human immunodeficiency virus-infected persons. J Infect Dis 170:1009–1013. doi:10.1093/infdis/170.4.1009. 7930696

[18] Davey RT, Boenning CM, Herpin BR, Batts DH, Metcalf JA, Wathen L, Cox SR, Polis MA, Kovacs JA, Falloon J, Walker RE, Salzman N, Masur H, Lane HC. 1994. Use of recombinant soluble Cd4 pseudomonas exotoxin, a novel immunotoxin, for treatment of persons infected with human-immunodeficiency-virus. J Infectious Diseases 170:1180–1188. doi:10.1093/infdis/170.5.1180. 7963711

[19] Ballantyne A, Dhillon S. 2013. Trastuzumab emtansine: first global approval. Drugs 73:755–765. doi:10.1007/s40265-013-0050-2. 23620199

[20] Chen W, Feng Y, Prabakaran P, Ying T, Wang Y, Sun J, Macedo CD, Zhu Z, He Y, Polonis VR, Dimitrov DS. 2014. Exceptionally potent and broadly cross-reactive, bispecific multivalent HIV-1 inhibitors based on single human CD4 and antibody domains. J Virol 88:1125–1139. doi:10.1128/JVI.02566-13. 24198429 PMC3911630

[21] von Minckwitz G, Huang CS, Mano MS, Loibl S, Mamounas EP, Untch M, Wolmark N, Rastogi P, Schneeweiss A, Redondo A, Fischer HH, Jacot W, Conlin AK, Arce-Salinas C, Wapnir IL, Jackisch C, DiGiovanna MP, Fasching PA, Crown JP, Wulfing P, Shao Z, Rota Caremoli E, Wu H, Lam LH, Tesarowski D, Smitt M, Douthwaite H, Singel SM, Geyer CE, Jr, Investigators K. 2019. Trastuzumab emtansine for residual invasive HER2-Positive Breast Cancer. N Engl J Med 380:617–628. doi:10.1056/NEJMoa1814017. 30516102

[22] Wang X, Cao M, Wu Y, Xu W, Wang Q, Ying T, Lu L, Jiang S. 2021. Synergistic effect by combining a gp120-binding protein and a gp41-binding antibody to inactivate HIV-1 virions and inhibit HIV-1 infection. Molecules 26:1964. doi:10.3390/molecules26071964. 33807292 PMC8036483

[23] Barok M, Joensuu H, Isola J. 2014. Trastuzumab emtansine: mechanisms of action and drug resistance. Breast Cancer Res 16:209. doi:10.1186/bcr3621. 24887180 PMC4058749

[24] Erickson HK, Park PU, Widdison WC, Kovtun YV, Garrett LM, Hoffman K, Lutz RJ, Goldmacher VS, Blattler WA. 2006. Antibody-maytansinoid conjugates are activated in targeted cancer cells by lysosomal degradation and linker-dependent intracellular processing. Cancer Res 66:4426–4433. doi:10.1158/0008-5472.CAN-05-4489. 16618769

[25] Hafeez U, Parakh S, Gan HK, Scott AM. 2020. Antibody-Drug Conjugates for Cancer Therapy. Molecules 25:4764. doi:10.3390/molecules25204764. PMC7587605 33081383

[26] Sogaard OS, Graversen ME, Leth S, Olesen R, Brinkmann CR, Nissen SK, Kjaer AS, Schleimann MH, Denton PW, Hey-Cunningham WJ, Koelsch KK, Pantaleo G, Krogsgaard K, Sommerfelt M, Fromentin R, Chomont N, Rasmussen TA, Ostergaard L, Tolstrup M. 2015. The Depsipeptide Romidepsin Reverses HIV-1 Latency In Vivo. PLoS Pathog 11:e1005142. doi:10.1371/journal.ppat.1005142. 26379282 PMC4575032

[27] Chen X, Zaro JL, Shen WC. 2013. Fusion protein linkers: property, design and functionality. Adv Drug Deliv Rev 65:1357–1369. doi:10.1016/j.addr.2012.09.039. 23026637 PMC3726540

[28] Platt EJ, Wehrly K, Kuhmann SE, Chesebro B, Kabat D. 1998. Effects of CCR5 and CD4 cell surface concentrations on infections by macrophagetropic isolates of human immunodeficiency virus type 1. J Virol 72:2855–2864. doi:10.1128/JVI.72.4.2855-2864.1998. 9525605 PMC109730

[29] Lu L, Pan C, Li Y, Lu H, He W, Jiang S. 2012. A bivalent recombinant protein inactivates HIV-1 by targeting the gp41 prehairpin fusion intermediate induced by CD4 D1D2 domains. Retrovirology 9:104. doi:10.1186/1742-4690-9-104. 23217195 PMC3531269

[30] Su S, Ma Z, Hua C, Li W, Lu L, Jiang S. 2017. Adding an artificial tail-anchor to a peptide-based HIV-1 fusion inhibitor for improvement of its potency and resistance profile. Molecules 22:1996. doi:10.3390/molecules22111996. PMC6150406 29156603

[31] Pan C, Cai L, Lu H, Lu L, Jiang S. 2011. A novel chimeric protein-based HIV-1 fusion inhibitor targeting gp41 glycoprotein with high potency and stability. J Biol Chem 286:28425–28434. doi:10.1074/jbc.M111.241992. 21690094 PMC3151085

[32] Pedro KD, Henderson AJ, Agosto LM. 2019. Mechanisms of HIV-1 cell-to-cell transmission and the establishment of the latent reservoir. Virus Res 265:115–121. doi:10.1016/j.virusres.2019.03.014. 30905686 PMC6467521

[33] Pincus SH, Song K, Maresh GA, Hamer DH, Dimitrov DS, Chen W, Zhang MY, Ghetie VF, Chan-Hui PY, Robinson JE, Vitetta ES. 2017. Identification of human anti-HIV gp160 monoclonal antibodies that make effective immunotoxins. J Virol 91:e01955-16. doi:10.1128/JVI.01955-16. 27852851 PMC5244328

[34] Cohn LB, Chomont N, Deeks SG. 2020. The biology of the HIV-1 latent reservoir and implications for cure strategies. Cell Host Microbe 27:519–530. doi:10.1016/j.chom.2020.03.014. 32272077 PMC7219958

[35] Shan L, Deng K, Shroff NS, Durand CM, Rabi SA, Yang HC, Zhang H, Margolick JB, Blankson JN, Siliciano RF. 2012. Stimulation of HIV-1-specific cytolytic T lymphocytes facilitates elimination of latent viral reservoir after virus reactivation. Immunity 36:491–501. doi:10.1016/j.immuni.2012.01.014. 22406268 PMC3501645

[36] Hashemi P, Barreto K, Bernhard W, Lomness A, Honson N, Pfeifer TA, Harrigan PR, Sadowski I. 2018. Compounds producing an effective combinatorial regimen for disruption of HIV-1 latency. EMBO Mol Med 10:160–174. doi:10.15252/emmm.201708193. 29246970 PMC5838563

[37] Wei DG, Chiang V, Fyne E, Balakrishnan M, Barnes T, Graupe M, Hesselgesser J, Irrinki A, Murry JP, Stepan G, Stray KM, Tsai A, Yu H, Spindler J, Kearney M, Spina CA, McMahon D, Lalezari J, Sloan D, Mellors J, Geleziunas R, Cihlar T. 2014. Histone deacetylase inhibitor romidepsin induces HIV expression in CD4 T cells from patients on suppressive antiretroviral therapy at concentrations achieved by clinical dosing. PLoS Pathog 10:e1004071. doi:10.1371/journal.ppat.1004071. 24722454 PMC3983056

[38] Leth S, Schleimann MH, Nissen SK, Højen JF, Olesen R, Graversen ME, Jørgensen S, Kjær AS, Denton PW, Mørk A, Sommerfelt MA, Krogsgaard K, Østergaard L, Rasmussen TA, Tolstrup M, Søgaard OS. 2016. Combined effect of Vacc-4x, recombinant human granulocyte macrophage colony-stimulating factor vaccination, and romidepsin on the HIV-1 reservoir (REDUC): a single-arm, phase 1B/2A trial. Lancet HIV 3:e463-72–e472. doi:10.1016/S2352-3018(16)30055-8. 27658863

[39] Pincus SH, Song K, Maresh GA, Frank A, Worthylake D, Chung HK, Polacino P, Hamer DH, Coyne CP, Rosenblum MG, Marks JW, Chen G, Weiss D, Ghetie V, Vitetta ES, Robinson JE, Hu SL. 2017. Design and in vivo characterization of immunoconjugates targeting HIV gp160. J Virol 91doi:10.1128/JVI.01360-16. PMC5244325 27795412

[40] Sadraeian M, Guimaraes FEG, Araujo APU, Worthylake DK, LeCour LJ, Pincus SH. 2017. Selective cytotoxicity of a novel immunotoxin based on pulchellin A chain for cells expressing HIV envelope. Sci Rep 7:7579. doi:10.1038/s41598-017-08037-3. 28790381 PMC5548917

[41] Scotti L, Filho FJBM, de Moura RO, Ribeiro FF, Ishiki H, da Silva MS, Filho JMB, Scotti MT. 2016. Multi-target drugs for neglected diseases. Curr Pharm Des 22:3135–3163. doi:10.2174/1381612822666160224142552. 26907943

[42] Su X, Wang Q, Wen Y, Jiang S, Lu L. 2020. Protein- and peptide-based virus inactivators: inactivating viruses before their entry into cells. Front Microbiol 11:1063. doi:10.3389/fmicb.2020.01063. 32523582 PMC7261908

[43] Brun MP, Gauzy-Lazo L. 2013. Protocols for lysine conjugation. Methods Mol Biol 1045:173–187. p doi:10.1007/978-1-62703-541-5_10. 23913147

[44] Chiang ZC, Chiu YK, Lee CC, Hsu NS, Tsou YL, Chen HS, Hsu HR, Yang TJ, Yang AS, Wang AH. 2020. Preparation and characterization of antibody-drug conjugates acting on HER2-positive cancer cells. PLoS One 15:e0239813. doi:10.1371/journal.pone.0239813. 32986768 PMC7521679

[45] Abedi M, Cohan RA, Mahboudi F, Faramarzi MA, Fazel R, Damavandi N, Ardestani MS, Davami F. 2019. Novel trastuzumab-DM1 conjugate: synthesis and bio-evaluation. J Cell Physiol 234:18206–18213. doi:10.1002/jcp.28453. 30854662

[46] Huang JH, Kang BH, Ishida E, Zhou TQ, Griesman T, Sheng ZZ, Wu F, Doria-Rose NA, Zhang BS, McKee K, O'Dell S, Chuang GY, Druz A, Georgiev IS, Schramm CA, Zheng AQ, Joyce MG, Asokan M, Ransier A, Darko S, Migueles SA, Bailer RT, Louder MK, Alam SM, Parks R, Kelsoe G, Von Holle T, Haynes BF, Douek DC, Hirsch V, Seaman MS, Shapiro L, Mascola JR, Kwong PD, Connors M. 2016. Identification of a CD4-binding-site antibody to HIV that evolved near-pan neutralization breadth. Immunity 45:1108–1121. doi:10.1016/j.immuni.2016.10.027. 27851912 PMC5770152

[47] Yang W, Sun Z, Hua C, Wang Q, Xu W, Deng Q, Pan Y, Lu L, Jiang S. 2018. Chidamide, a histone deacetylase inhibitor-based anticancer drug, effectively reactivates latent HIV-1 provirus. Microbes Infect 20:626–634. doi:10.1016/j.micinf.2017.10.003. 29126877

