# Supplementary figures and images for: A Toxin-Conjugated Recombinant Protein Targeting gp120 and gp41 for Inactivating HIV-1 Virions and Killing Latency-Reversing Agent-Reactivated Latent Cells
Source: mBio. 2022 Jan 18;13(1):e03384-21. doi: 10.1128/mbio.03384-21 (PMC8764533; doi:10.1128/mbio.03384-21)

## SUPPLEMENTARY MATERIAL

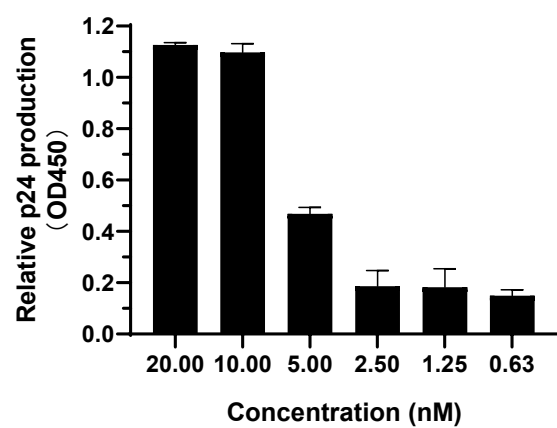

**Fig. S1**

Supplement: FIG S1 [file mbio.03384-21-sf001.pdf]

## SUPPLEMENTARY MATERIAL

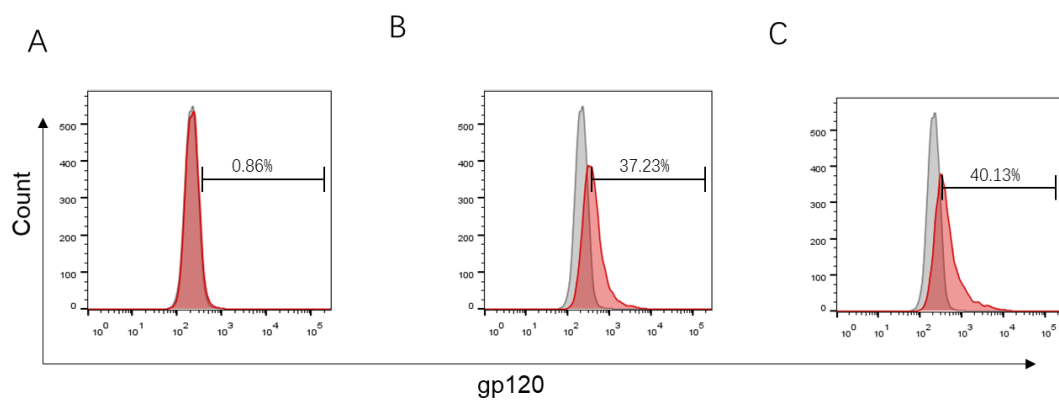

**Figure S2**

Supplement: FIG S2 [file mbio.03384-21-sf002.pdf]

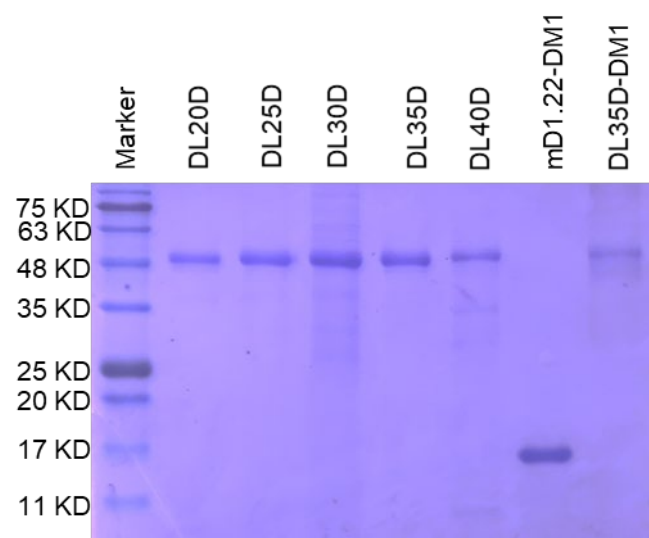

FIG. S3

Supplement: FIG S3 [file mbio.03384-21-sf003.pdf]
